# Personalising Supportive Healthcare for Immune-Mediated Inflammatory Disorders: A Qualitative Exploration of Patient Needs and Behaviours Based on the Subjective Health Experience Model

**DOI:** 10.5334/ijic.8965

**Published:** 2025-11-07

**Authors:** Tessa S. Folkertsma, Greetje J. Tack, Robert M. Vodegel, Sjaak Bloem, Aad R. Liefveld, Maya Schroevers, Reinhard Bos

**Affiliations:** 1 Department of Gastroenterology, Frisius MC, Henri Dunantweg 2, 8934 AD, Leeuwarden, The Netherlands; 2 Department of Dermatology, Frisius MC, Henri Dunantweg 2, 8934 AD, Leeuwarden, The Netherlands; 3 Department of Rheumatology, Frisius MC, Henri Dunantweg 2, 8934 AD, Leeuwarden, The Netherlands; 4 Center for Marketing & Supply Chain Management, Nyenrode Business University, Straatweg 25 3621 BG, Breukelen, The Netherlands; 5 Link2Trials, Neptunusstraat 16, 1223 HL, Hilversum, The Netherlands; 6 Department of Health Psychology, University Medical Center Groningen, Hanzeplein 1, 9713 GZ, Groningen, The Netherlands

**Keywords:** immunology, Subjective Health Experience, personalised supportive healthcare, treatment guidelines, integrated care, team-based healthcare

## Abstract

Current insights into how to personalise supportive care for patients with immune-mediated inflammatory disorders (IMIDs) remain limited. Enhancing supportive care can significantly improve patients’ quality of life and overall healthcare. The Subjective Health Experience (SHE) model offers a practical framework for segmenting patients based on disease acceptance and control, potentially guiding tailored supportive care.

This qualitative study had two aims: explore patient characteristics (behaviours, questions, and needs) within each SHE Segment; and determine required supportive care per Segment by identifying the *what* (specific types of supportive healthcare) and the *how* (approach of healthcare delivery).

Group discussions and individual interviews were conducted with 19 healthcare professionals in rheumatology, gastroenterology, and dermatology, and 18 patients diagnosed with rheumatoid arthritis/spondyloarthritis, Crohn’s disease/ulcerative colitis, or psoriasis/hidradenitis suppurativa.

Findings revealed consistent patterns across IMIDs regarding healthcare needs. Patients emphasised the importance of attention and acknowledgement, while healthcare professionals focused on structure and planning. Detailed Segment descriptions supported development of a structured framework aligning supportive care types and delivery approaches with each SHE Segment.

Overall, these results support the SHE framework as a guidance for coordinating supportive care across conditions, professionals, and care levels, enhancing its operational use in IMID care to improve personalisation and continuity.

## Introduction

The quality of life (QoL) of individuals with immune-mediated inflammatory disorders (IMIDs) is significantly affected not only by physical and biomedical complications, but also by psychological and psychosocial challenges. Disease-specific complications include joint deformities, uveitis, and osteoporosis for rheumatoid arthritis (RA) and spondyloarthritis (SpA) [1,2]; malnutrition, colorectal cancer, stenosis, and fistulas for inflammatory bowel disease (IBD; Crohn’s disease and ulcerative colitis) [3,4,5,6,7,8]; and arthritis, cardiovascular diseases, and secondary bacterial infections for psoriasis (PsO) and hidradenitis suppurativa (HS) [9,10,11]. Psychological and psychosocial issues, such as depression, anxiety, stigmatisation, and social withdrawal, often overlap across these disorders [12,13,14]. In addition to shared psychosocial impacts, IMIDs frequently co-occur and are characterised by overlapping pathophysiological mechanisms, chronicity, and treatment demands [15].

Besides appropriate medical treatment, it is essential to consider how individuals experience their own health [16]. Combining these experiences with biomedical perspectives can streamline diagnosis, refine treatment plans, improve continuity of care, enhance patient adherence, and raise patient-reported satisfaction with care delivery [17]. Insights into health perception can also inform supportive strategies tailored to the patient’s unique context, and needs [18].

Contemporary healthcare paradigms are progressively gravitating toward ‘tailored care’, the right support at the right time, in the right place [19]. This shift is driven by rising healthcare demand due to the increased prevalence of chronic (immune-mediated inflammatory) disorders [20,21,22], and personnel shortages [23]. Efficient resource deployment and active patient engagement are crucial for the treatment of IMIDs.

**Figure 1:**
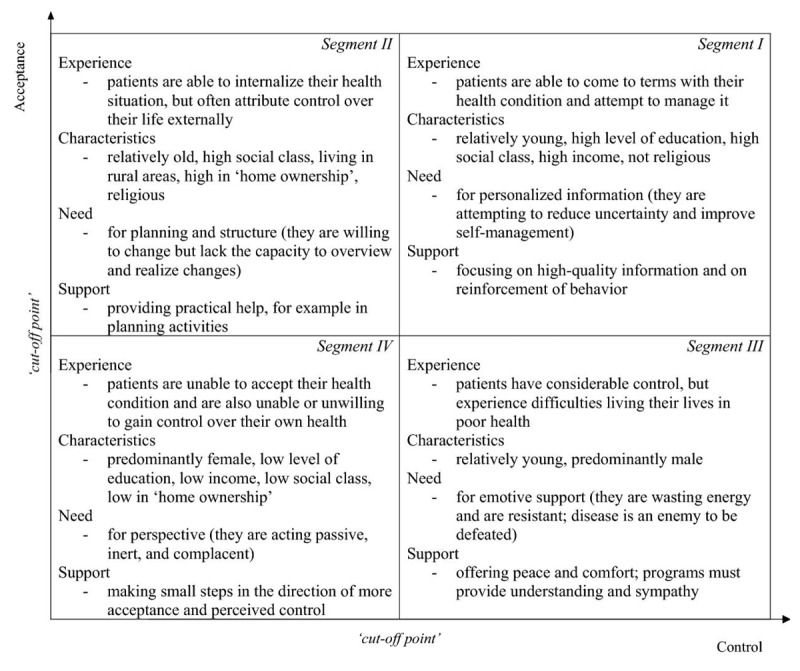
SHE model based on Bloem, S. & Stalpers [17].

Bloem and Stalpers developed a model that offers insights into the subjective health experience (SHE) of patients and their associated needs [18]. These needs define healthcare interventions used alongside conventional treatments in the context of tailored care. They define SHE as: “An individual’s experience of physical and mental functioning while living his life the way he wants to, within the actual constraints and limitations of individual existence.” [18, p. 8]. Two psychological determinants, acceptance and perceived control, are central to this concept. A higher level of acceptance – the ability to integrate health status into daily life – and perceived control – the extent to which patients perceive opportunities to improve their condition – are both linked to more positive health perceptions. These constructs are measured using three items each, and together form the basis of a segmentation model that prescribes who needs which support, and when. This establishes a practical framework for initiating tailored care (Figure 1). The SHE model is inherently dynamic, reflecting the evolving nature of health perception and support needs. Consequently, appropriate care modalities should exhibit adaptability over time.

Several studies have been conducted using the SHE model. For instance, the Segments within this model have been further differentiated based on demographic, and socio-economic variables [16], and in relation to vitality within older individuals [24]. In addition, health perceptions have been mapped across diverse disease domains [25], and the empirical relationship between health perception and health behaviour has been established [26]. Furthermore, in two distinct studies (cross-sectional and longitudinal), the model was validated for an IBD cohort [27,28]. Extending its application to supportive healthcare for IMIDs represents a logical, and valuable, next step, given the complex and overlapping needs of this patient group.

While the SHE model offers a guide for tailored care, it has not yet been applied to the specific supportive care needs of patients with IMIDs or their healthcare providers. Given the overlap in pathogenesis, treatment burden, chronicity, and co-occurrence of IMIDs, their supportive care needs are hypothesised to follow similar patterns. This aligns with earlier calls for more integrated, multidisciplinary management of these conditions [15]. This qualitative study has two aims. First, it seeks to generate nuanced insights into patient characteristics—specifically behaviours, questions, and needs—across the four SHE segments, in order to refine the personalised supportive care framework. These insights are intended to improve alignment between patients’ lived experiences and the professional roles involved in their care. Second, it explores how the SHE model can differentiate both the *what* (specific types of supportive healthcare) and the *how* (approach of healthcare delivery) of supportive care for patients with IMIDs. The findings aim to inform structured, and segment-based, delivery of supportive care that can enhance interdisciplinary coordination, clarify roles, and strengthen continuity within integrated care systems.

## Method

This study is part of an extensive research project aimed at offering and monitoring more personalised care related to IMIDs and treatments based on Patient Reported Outcome Measures (PROMs). It also aimed to enhance collaboration among the departments of gastro-enterology, rheumatology, and dermatology. A proposal for this research was submitted to the local Medical Ethics Committee at Frisius MC in May 2020 and was approved, being classified as not subject to the Medical Research Involving Human Subjects Act (non-WMO in Dutch). The recruitment period for this study was between 1 May 2020 and 18 April 2022.

### Study design

Collaborative discussions were initiated with both healthcare professionals and patients from three different disciplines, namely gastroenterology, rheumatology, and dermatology, at Frisius MC, a prominent regional hospital in the Netherlands.

The study used qualitative methods with ethnographic elements and phenomenological inquiry to gain in-depth insights into the subjective experiences of healthcare professionals and patients [29,30]. The study was grounded in the constructivist/interpretivist paradigm, focusing on understanding the complex world of lived experiences from the perspective of those who experience them [31]. This paradigm emphasises the co-construction of data through researcher–participant interactions, acknowledging multiple realities and the importance of context in understanding experiences.

The study was guided by the SHE model, which structures the exploration of supportive care. Both healthcare professionals and patients with an IMID characterised the behaviour, questions, and needs of patients from the different Segments of the SHE model. For the patient groups, discussions also delved into their disease perceptions. With both healthcare professionals and patients, an inventory was drawn up of the types of supportive healthcare offered by the hospital treatment teams, and the approach of this healthcare delivery was subsequently allocated to one of the study’s four predefined Segments.

### Participants and procedure

Participants were recruited via purposive and convenience sampling. Healthcare professionals were eligible if they treated patients with IMIDs within rheumatology, gastroenterology, or dermatology. Patients were eligible if they had been treated at Frisius MC within the prior six months.

Three physicians, one from each discipline, undertook the recruitment process, verbally inviting healthcare professionals and extending email invitations to patient participants. Participation was voluntary, and no compensatory incentives were disbursed. All participants gave written informed consent.

Focus groups were held with professional teams from all three disciplines, as well as with patients diagnosed with rheumatoid arthritis/spondyloarthritis and IBD (Crohn’s disease/ulcerative colitis) at Frisius MC. Due to COVID-19, patients with psoriasis/hidradenitis suppurativa were interviewed individually via Microsoft Teams. Sessions were moderated by an experienced, independent qualitative researcher.

### Guided discussion protocols

The semi-structured discussions followed a predefined checklist tailored to explore behaviours, questions, and supportive care needs across the four SHE Segments. Sessions began with an explanation of the study purpose and concluded with next steps. Segment descriptions developed with healthcare professionals were used to guide discussions with patients. Interviews lasted around 30 minutes; group discussions around 2 hours. Tables 1 and 2 outline the interview structure and outputs.

**Table 1:** Structure and outputs of group discussions with healthcare professionals.


FOCUS AREAS	OUTPUT

Short explanation of the SHE model – Describe each Segment individually in terms of behaviours, questions, and needs. Constantly refer to your own professional experience, envisioning individuals who typically fit within each Segment. Segments were defined either in small groups or individually, and the results were subsequently shared with the larger group.	Descriptions of the Segments, resulting in a framework for differentiating healthcare modalities.

Inventory of provided services – What services do you offer to support and guide patients? Information was collected collaboratively and documented on a flipchart.	An inventory of available services was compiled.

Assigning support to Segments (previous descriptions were visible) – What type of support is typically suited to each Segment? A group discussion was conducted to determine this.	Consensus was reached through group discussion on how to allocate support across Segments.


**Table 2:** Structure and outputs of group discussions and individual interviews with patients with IMIDs.


FOCUS AREAS	OUTPUT

Top-of-Mind characteristics – Describe your own condition and health experiences. Including emotional responses to the diagnosis, their need for support, and the impact of the condition on their daily life. Outcome was documented on flip charts. – Select the three most significant words for you and describe what they mean in your daily life. This was an individual task, followed by a group discussion.	Reflection on what the condition meant for the individual in their daily life; the aspects that played a role in this.

Health Ladder (Visual ladder with 11 Steps, brief explanation) – Describe an individual at the bottom and top of their health ladder. What does the person need to ascend the ladder? What does the hospital provide? This was discussed collectively.	Insights into behaviour, questions, and needs; what was required; what was offered. (Note: High on the ladder corresponds with Segment 1; low on the ladder corresponds with Segment 4)

Explanation of segmentation model (short video and brief description) – For each Segment (visual provided with a brief description based on input from healthcare professionals), what services does Frisius MC offer for this individual? This was an individual task, followed by group discussion. Note: In TEAM interviews, words were typed and displayed on the screen; the video was not shown.	Insights into forms of support per Segment.


### Analytical approach

Interview transcripts were systematically analysed using the matrix method proposed by Groenland [32]. A matrix was devised, with rows for healthcare professionals and patients, and columns representing specific interview questions. Direct participant responses were transcribed and populated in this matrix.

In a subsequent phase, a thematic matrix was established to categorise and consolidate themes across interview questions. Through tallying, predominant themes were identified, highlighting variances and commonalities within and between participant groups. The analysis was conducted manually.

Data saturation was considered reached when consecutive interviews yielded no new themes and when variations within existing themes were fully explored across all participant groups.

To ensure accuracy and credibility, the results were presented to a selection of healthcare professionals for member checking, accommodating their feedback to confirm correct interpretation. Additionally, the methods section of this article is reported according to the Standards for Reporting Qualitative Research guidelines [33].

This analysis provided detailed insights into the behaviours, questions, and needs specific to patients with IMIDs. These insights guided how tailored care strategies provided by the hospital, could be effectively distributed across the Segments. The matrix analysis facilitated the display, interpretation, and evaluation of findings, as suggested by Averill [34], enhancing the development of evidence in qualitative research.

## Results

### Participant characteristics

Nineteen healthcare professionals participated: eight from the RA/SpA team (three physicians, five nurses), six from the IBD team (one physician, five nurses), and five from the PsO/HS team (one physician, four nurses). Gender, age, and years of professional experience varied.

Eighteen patients took part: six with RA/SpA (4 RA, 2 SpA), seven with IBD (three Crohn’s disease, four ulcerative colitis), and five with PsO/HS (three PsO, two HS). Ten were female and eight male. Ages ranged from 22 to 74 years, and disease duration from 2 to 50 years.

### Behavioural patterns, questions, and needs

The four Segments of the SHE model, based on levels of acceptance and perceived control, structured the findings. Segment 1 reflects high acceptance and high control; Segment 2, high acceptance and low control; Segment 3, low acceptance and high control; and Segment 4, low acceptance and low control. Each Segment was further analysed using three categories: cognition and behaviour, questions and dilemmas, and specific support needs. These categories were derived from semi-structured interviews and group discussions with healthcare professionals and patients diagnosed with an IMID (RA/SpA, IBD, or PsO/HS). Due to substantial thematic overlap across participant groups, the results were consolidated into a single table. Table 3 presents the combined Segment characteristics. Lines indicate relationships between categories within each Segment, signifying thematic interdependence.

**Table 3:** SHE Segment characteristics based on interviews and discussions with both healthcare professionals and patients (RA/SpA, IBD, PsO/HS).


COGNITION AND BEHAVIOUR	QUESTIONS AND DILEMMAS	SPECIFIC SUPPORT NEEDS

**Segment 1** high acceptance and high control		

Attaches great importance to factual information	“What are reliable sources of information?”	**“I need certainty”** Desire for validation of one’s own approach

Keeps informed		

Keeps up with developments regarding the condition	“What new developments are relevant to me?”	

Prepares for healthcare visits	“What diagnostic or treatment options am I unaware of?”	Shared decision-making
Copes easily with setbacks	“How can I manage or deal with the fluctuating course of my condition?”	Feeling heard and acknowledged by healthcare professionals

**Segment 2** high acceptance and low control		

Seeks guidance to better cope with the condition	“How can I gain control?”	**“I need structure”** Support to regain control over the situation Managing matters (themselves) with support from others

Attempts to prepare for consultations	“Am I doing the right things?”	Feeling heard and acknowledged by healthcare professionals

Consults multiple healthcare professionals	“What (lifestyle) adjustments will help me manage my condition?”	Comprehensive overview of disease progression, treatment and support options.

Open attitude towards the treatment team	“Am I doing enough?”	

**Segment 3** low acceptance and high control		

Questions why this happens to them specifically	“How can I learn to live with this?”	**“I need tranquillity”**

Wants to keep things the way they are	“How can I (re)organise my life?”	Support with coping and self-image– active role for healthcare professional

Tends to blame themselves	“How do I avoid social isolation?”	

Struggles with shame about their illness	“How can I best cope with others’ reactions?”	Support for family and friends during the disease process

Does not readily express emotions to healthcare professionals	“How can I discuss it with others ?”	Examples of others with the same condition

Primarily seeks support from family and friends	“How do I maintain an (intimate) relationship with my partner?”	Someone who listens

**Segment 4** low acceptance and low control		

Feels unable to break out of their situation	“How can I learn to live with this condition?”	**“I need perspective”**

Prefers to let everything sink in before acting		

Is undecided about treatment options but does want support	“What is the next step?”	Small, concrete tasks and simple advice

Engages in discussions under pressure from others	“Who can be trusted?”	Feeling heard and acknowledged by healthcare professionals

Demands a lot of attention from healthcare professionals	“What exactly can be done?”	Step-by-step guidance and direction Active role for healthcare professionals

Is pessimistic and tends to blame others	“Is this never going to pass?”	


Legend: Lines are used to indicate relationships between the three characteristics.

In Segment 1 (high acceptance, high control), patients are defined by their ability to cope with the consequences of chronic inflammatory diseases. They display a high degree of openness when discussing their conditions with others and handle comments in a thoughtful manner. Overall, they strive to integrate the condition into daily life and engage in activities matching their capabilities. A key aspect of their approach is an intrinsic motivation to remain well-informed. They proactively seek new information, often bringing critical questions to consultations. This Segment prefers active involvement in decisions about treatment and support, identifying with the concept of self-management, and seeks external validation from healthcare professionals and loved ones to affirm their adaptive approach.

In Segment 2 (high acceptance, low control), patients exhibit a positive attitude towards their condition and actively work to optimise well-being. Like those in Segment 1, they integrate their conditions and symptoms into daily life. However, they often lack a comprehensive understanding of available interventions and more frequently question whether they are taking the right steps to improve QoL. Providing structured support and guidance is essential to help these patients manage their conditions effectively.

Segment 3 (low acceptance, high control) includes patients who struggle to accept their disease and adapt to their condition. This Segment often consists of patients recently diagnosed or whose circumstances have recently deteriorated. They may seek support from loved ones but also experience feelings of shame. They have many questions about managing their altered situation, both personally and socially. Immediate assistance in coping with the condition and self-perception, aimed at regaining balance, is crucial.

Segment 4 (low acceptance, low control) describes patients who have lost hope and see no way to cope with the impact of their disease on QoL. Pervasive pain, fatigue, and other symptoms are significant impediments. They tend to withdraw from their social environment, driven by feelings of helplessness and shame. Managing comments, whether well-intentioned or not, is challenging, leading to isolation, misunderstanding, and melancholy. Their passive disposition makes motivating them towards proactive measures difficult. Central to their concerns is the question of ‘what’s next?’, which becomes more pressing as they face numerous lifestyle guidelines. Incremental progress through small tasks and consistent guidance may offer a path forward. Engaging in activities and seeing results may help restore a sense of purpose.

Overall, the Segment characteristics across the six IMIDs displayed considerable overlap. Despite apparent distinctions between these conditions, notable similarities exist in patients’ challenges, resulting in similar behaviours, questions, and needs. For example, all patients consistently mentioned pain, while fatigue was reported by both RA/SpA and IBD patients. Both symptoms greatly impact daily activities. Furthermore, patients from all disorders struggle with shame, though its manifestation varies—for example, reluctance to ask for help (RA/SpA); embarrassment due to frequent, urgent toilet use and faecal incontinence (IBD); and feeling filthy and perceived as such by others (PsO/HS).

### Tailored care

In Table 4, a comprehensive overview outlines the various forms of hospital-provided support for each Segment of the SHE model, as identified and categorised by healthcare professionals and patients (RA/SpA, IBD, and PsO/HS). The types of support discussed in the interviews were explicitly related to specific IMIDs but are generalised in the overview, except where support was uniquely applicable to a particular condition. During the discussions, and prompted by the moderator, additional criteria were defined to refine the categorisation of supportive care modalities. Participants distinguished between the *what*—specific types of supportive healthcare—and the *how*—approach of healthcare delivery. This distinction led to the development of a structured framework that lists the types of supportive care offered by healthcare professionals (bold text in Table 4) alongside the methods tailored to each SHE Segment (non-bolded text in the middle column, validated by checkmarks in the rightmost column).

**Table 4:** Tailored care framework based on discussions with both healthcare professionals and patients (RA/SpA, IBD, PsO/HS).


CATEGORY	SUPPORTIVE INTERVENTION	SHE SEGMENT			

**Consult with healthcare professional**	**Validation and building trust**	**1**	**2**	**3**	**4**

	– Adapt communication and tone to patient’s needs	√	√		
	○ Patient initiates interaction and articulates needs	√			
	○ Provide a clear overview when necessary		√		
	– Offer tranquillity and perspective			√	√
	○ Help foster acceptance and promotes self-awareness			√	
	○ Provide a structured treatment timeline				√

	**Attention and active listening**				

	– Maintain a connection over time	√	√		
	○ Provide low-threshold access to contact (e.g. remote options)	√	√		
	○ Offer positive reinforcement (“You’re doing well”)	√			
	○ Continuously reassure availability of support		√		
	– Be present			√	√
	○ Demonstrate openness and shows understanding (empathy and compassion) for the individual			√	
	○ Acknowledge complaints and dedicate time				√

**Discussion with healthcare professional**		1	2	3	4

	– Share decision-making on the treatment plan	√	√		
	○ Reciprocal thinking and dialogue	√			
	○ Structure information and planning		√		
	– Explore treatment options based on patient preferences (guided decision-making)			√	√
	○ Initially focus on the condition – subsequently on the treatment plan			√	
	○ Gradually involve patient in the treatment plan and future possibilities				√
	– Routine-based, less frequent consultations	√			
	– Provide an overview (role for nurse)		√		
	– Discuss mental wellbeing alongside physical symptoms			√	√
	○ Early referral to appropriate professionals (e.g., psychologist, dietician)				√

**Information (transfer) and instructions**	**From diagnosis to medication**	1	2	3	4

	**Orally**				
	– Explain concise	√	√		
	– Explain in more detail		√	√	
	– Explain step-by-step, with repetition if necessary			√	√
	**Print/digital information and reliable sources**				
	– Provide (relevant to patient context) and hand over				
	○ New developments	√			
	○ Overviews		√		
	– Provide, review together, and hand over				
	○ In sections			√	√
	○ Aftercare, e.g., understanding the information, follow-up calls				√
	**Information sessions (meetings)**				
	– Inform about	√	√		
	– Encourage attendance explicitly			√	√
	– Experts by experience at the forefront as inspiration		√	√	√

**Channels and frequency**	**Contact methods and intensity**	1	2	3	4

	**Type of channel**				
	– More remote: e-mail only	√			
	– Less remote: phone (video call), email		√		
	– More direct contact: hybrid, video call (phone), face-to-face			√	
	– Direct contact: face-to-face preferred (with digital support as needed)				√
	– Department (reception – availability 8:00 – 18:00)	√	√	√	√
	**Frequency of contact**				
	– Limited (annually), tailored to the patients’ needs	√	√		
	– Intensive			√	√

**Third party support**		1	2	3	4

	**Referral to another professional** (e.g., general practitioner, dietician, physiotherapist, psychologist, social worker, home care, sexologist, rehabilitation doctor (not PsO), alternative medicine (mentioned by patients))				
	– Referral channel				
	○ Written	√	√	√	
	○ Verbal (phone)				√
	**Caregiver**				
	– Is welcome (during consult)	√	√		
	○ As a partner	√			
	○ As a supporter (external commitment)		√		
	– Actively involve (in consultation)			√	√
	○ As an ally			√	
	○ As a motivator				√
	**Patients’ association**				
	– Raise awareness (reliable source)	√	√		
	– Discuss possibilities (e.g., peers)			√	√

**Digital support** (also see information transfer)		1	2	3	4

	**Applications and programmes** (e.g., *IBDcoach* (a mobile application designed to support patients at home) or lifestyle interventions such as *Nederland in Beweging* (a Dutch television programme that promotes physical activity).				
	– Inform about	√	√		
	– Discuss along with the caregiver			√	√
	**Podcasts**				
	– Inform about	√	√		
	– Discus along with caregiver			√	√

**Promoting lifestyle and therapy adherence** (nutrition, movement, smoking and drinking, pregnancy, sun, vaccination)	**Support** mostly by nurse, referral if necessary	1	2	3	4

	– Highlight importance and provide an overview of behavioural rules and available programmes	√			
	– Encourage automate (new) behaviour by offering behavioural rules and available programmes		√		
	– Discuss behavioural rules and available programmes explicitly			√	√
	– Offer guided aftercare and follow-up				√


Legend: **bold** – mainly focused on *what* is offered; not bold – mainly focused on *how* it is offered. Numbers in the right column refer to Segments: Segment 1 – high acceptance, high control; Segment 2 – high acceptance, low control; Segment 3 – low acceptance, high control; Segment 4 – low acceptance, low control.

The participants, comprising healthcare professionals and patients, sometimes found it challenging or non-intuitive to allocate specific forms of support to a Segment. Initially, both groups often stated that universally relevant information is needed across Segments. However, some participants noted spontaneously that the method of delivery could vary. For example, providing information on explicit request was linked to Segment 1, while tailoring and structuring information was considered more suitable for Segment 2. More intensive engagement in discussing information was viewed as particularly beneficial for Segments 3 and 4.

Participants suggested that information could be for independent review at home or interactively discussed during consultations. Variations included structuring content, adjusting consultation time, and selecting appropriate communication channels before and during visits. These insights offered practical guidance for tailoring supportive care across Segments.

During discussions, particularly among healthcare professionals, it became clear that an individual’s acceptance of their health condition strongly influences the preferred supportive care strategy (*how*). Patients with higher acceptance (Segments 1 and 2) respond well to reactive care, often showing initiative and autonomy. Shared decision-making works effectively for this group. Conversely, those with lower acceptance (Segments 3 and 4) exhibits a lack of initiative and require proactive support and external guidance for organising care and decision-making, benefitting from ‘guided decision-making’ across disciplines and care levels., requiring external guidance for organising appropriate care and decision-making.

Healthcare professionals noted that patients in Segments 3 and 4 struggle more to integrate their condition into their lives. They emphasised the importance of focusing on the inherent implications of the disorder for the individual before discussing treatment modalities. The process of grief, acceptance, and active involvement is universally experienced by patients, regardless of their specific condition. Yet, this crucial process is often inadequately addressed by healthcare professionals, who tend to prioritise quantifiable aspects of disease activity during consultations.

When identifying suitable care for each Segment, patients emphasised the importance of ‘contact’ with healthcare professionals. The unanimous consensus among patients was that ‘recognition’ is essential for trust and long-term professional relationships. This need spanned treatment, medication, and physical and psychological support. Over half of patient respondents stressed that personalised recognition enhances mutual understanding and care quality.

Several illustrative examples from respondents highlighted the importance of trust-based consultations, including gastroenterology consultations about pregnancy and medication, rheumatology re-evaluations due to fatigue and reduced work capacity, and dermatology discussions on the emotional toll of visible skin lesions during summer. Across these scenarios, ‘recognition’ and ‘trust’ in healthcare professionals were pivotal in enabling effective healthcare provision.

Some patients described a sense of ‘vulnerability,’ especially with physicians, which could hinder open communication. Non-judgemental listening was seen as essential for patient-centred care and empathic understanding.

These findings informed a nuanced differentiation of care strategies across Segments (Table 4). In third-party support, healthcare professionals varied communication channels: written referrals were common in Segments 1–3, while telephone contact was reserved for Segment 4. Additionally, healthcare professionals delineated nuanced roles for caregivers of patients with IMIDs. In Segment 1, caregivers act as ‘equal partners,’ providing affirmation and encouragement, whereas in Segment 2, they serve as ‘supporters,’ helping patients adhere to appointments and acting as external commitments. In Segment 3, caregivers play the role of ‘empathisers’ who understand and engage in discussions, and in Segment 4, they function as ‘motivators’ facilitating incremental progress. Healthcare professionals should educate and guide caregivers accordingly during consultations.

Lastly, healthcare professionals noted the limited availability of digital support tools (data collected before the COVID-19 pandemic), suggesting a need for their development, ideally at a hospital-wide level.

As with patient characteristics, supportive care options across the six IMIDs showed almost complete overlap. Minor differences included the dermatology department rarely referring patients to rehabilitation, a practice more common in other specialties.

## Discussion

This study explored how the SHE model can be applied to personalise supportive care for patients with IMIDs. The findings showed that, despite differences between conditions, patients reported highly similar challenges, behaviours, and supportive needs. Consequently, both *what* specific types of supportive healthcare are offered and *how* it is delivered were comparable across IMIDs, supporting a cross-disciplinary care design that enhances quality and efficiency. Patients consistently valued attention, acknowledgement, and active listening, while healthcare professionals highlighted the need for structure and planning. Mapping supportive care modalities to the SHE Segments provided a practical framework to guide tailored care and strengthen integrated service delivery.

These insights highlight opportunities to refine supportive care by personalising pathways, detecting mismatches between needs and services, and identifying when unmet needs emerge.

Attention and active listening were particularly emphasised by patients, fostering recognition and trust. Scientific literature confirms these as strong indicators of the quality of interactions between healthcare professionals and end-users [35,36,37]. For instance, attention and active listening contribute to a multitude of favourable outcomes, including heightened patient motivation, reduced frequency of healthcare service utilisation, and elevated patient satisfaction [38,39,40]. While the importance of ‘attention’ is commonly emphasised, ‘listening’—especially unbiased listening without immediate solutions—is often underemphasised. Rogers developed targeted therapeutic methods to enhance active listening [41], while Van de Pol operationalised this through the ‘listening thermometer’, a practical tool for translating listening into acknowledgment with potential for clinical use [42].

All study participants exhibited a comprehensive understanding of the SHE segmentation model and its practical applicability, though they found it difficult to categorise support strictly by Segment. However, study discussions led to innovative ways of differentiating support, such as distinguishing between ‘providing’ and ‘discussing’ information with patients. These distinctions gave participants new tools for allocating support more precisely, and future research could expand this into a comprehensive list of such mechanisms. Interestingly, the utilisation of digital support was seldom mentioned by participants in any group. This may be attributed to limited availability (this study was conducted partially pre-COVID-19) and a lack of awareness among participants. As digital technology becomes increasingly important within tailored care, it will be crucial to focus on this area.

Comparable frameworks, such as COM-B, address behaviour through capability, opportunity, and motivation [43]. The SHE model, however, provides a distinct and practice-oriented advantage by segmenting patients according to acceptance and perceived control—two psychological determinants closely linked to health perception. This segmentation not only identifies specific types of supportive healthcare (*what*) but also determines the most appropriate delivery approach (*how*). By combining these elements, the SHE model bridges the gap between understanding a patient’s psychological profile and translating it into actionable, tailored interventions. This dual focus enables more precise alignment of resources, fosters interdisciplinary coordination, and supports continuity of care, making the SHE model particularly suited for integrated and personalised healthcare.

### Strengths and Limitations

A key strength of this study is its ecological validity, achieved through involving both healthcare professionals (physicians and nurses), and patients from six IMIDs across three disciplines. The resulting framework, based on behaviour, questions, and healthcare needs, offers a valuable resource for both inspiration and evaluation in designing and assessing supportive care.

Nonetheless, the study exhibits some limitations. The evaluation was confined to services provided by three treatment teams in a single peripheral healthcare institution. This single-centre design might limit generalisation to other settings. Similarly, initiatives not examined here may better meet the healthcare needs of this patient population. No triangulation with clinical or observational data was undertaken, as the study focused on participants’ subjective experiences and perceptions, which are not always directly observable or documented in clinical records. The study did not examine the qualitative aspects of the support provided, which may influence perceived care quality.

The perceived ‘vulnerability’ of patients, due to their sense of inferiority to healthcare providers, may have inhibited open dialogue during consultations and influenced their responses during the study’s interviews. Member checking was conducted only with healthcare professionals. Although planned with patients, follow-up attempts were unsuccessful due to the time elapsed after data collection. As a result, patient perspectives could not be directly validated, which may have introduced a higher risk of researcher bias or misrepresentation. Nonetheless, patient data were analysed inductively and discussed within a multidisciplinary research team to minimise bias.

### Practical implications

Implementation of this framework may face barriers, such as workforce time constraints and uneven digital infrastructure; however, its potential to enhance coordination, role clarity, and continuity of care within integrated healthcare systems is considerable. Leveraging this potential is particularly relevant given the significant overlap between IMIDs, which supports the creation of a cross-disciplinary supportive care plan. Such a plan could include sharing resources and referral networks, establishing interdisciplinary support teams for patients with overlapping conditions, developing shared digital platforms, organising joint information sessions for new patients, and forming multidisciplinary therapy groups. This level of collaboration has the potential to improve both care quality and treatment efficiency.

Operationalising the SHE model demonstrates that support can be differentiated into appropriate care in a relatively straightforward way. Integrating the model into hospital electronic health records would enable tailored guidance based on patients’ acceptance and control levels, supported by real-time monitoring and periodic reassessment. The electronic health records should include a list of available support options and record which patients receive which forms of support, allowing ongoing evaluation. Clear dashboards for both professionals and patients could help optimise care delivery, strengthen patients’ perception of health, and enhance overall quality of life. This forms the foundation for efficient, appropriate care. In addition to its clinical utility, the framework can also inform the work of professionals in coordinating patients with broader health and community resources. By segmenting patients’ needs, roles such as health coaches and link workers could more effectively connect individuals with suitable lifestyle, self-management, and social support services.

Further development and implementation of digital tools are essential. While the model’s validity and value have been demonstrated in manual application, digital integration is needed to ensure consistency and maximise impact [44]. Hospital innovation departments could lead this process across multiple specialties.

Although developed and tested in a clinical care setting, the benefits of tailored care also extend to clinical research. Clinical studies, while more controlled, rely on collaboration between healthcare professionals and patients to achieve successful outcomes. Yet, early drop-out rates average 25%, with nearly 70% of protocol deviations linked to non-adherence [45]. These challenges extend timelines, increase costs, reduce efficacy, and create missing data. Applying the SHE model to segment and tailor support could improve adherence, reduce drop-out, and enhance protocol compliance—making studies more efficient and cost-effective.

Overall, the structured application of the SHE model offers a practical tool for personalised support across disciplines. By guiding the allocation of supportive care, it can enhance coordination, clarify provider roles, and improve continuity within integrated care systems.

### Future Research

Future research should explore a broader range of clinical domains to identify disease-specific differences or trends across diverse healthcare settings and to demonstrate that the insights offered by the SHE profiles into support needs (*what* and *how*) can assist healthcare providers in determining the most effective way to support patients, regardless of discipline or level of care.

Research should also examine the dynamic nature of disease processes, including condition progression and treatment, and how patient behaviour and needs evolve over time, along with the implications for healthcare provision. Evaluating the effectiveness of healthcare interventions is vital, identifying which approaches are most effective, which are less so, and what modifications could improve outcomes.

Furthermore, patients with new-onset chronic IMIDs typically undergo processes of sorrow, understanding, acceptance, and lifestyle changes. Ultimately, using a general approach, the SHE model could facilitate adequate supportive treatment for patients grouped by segmentation, irrespective of their IMID type, and, in the longer term, its integration into care coordination workflows or digital care planning systems could further enhance both efficiency and patient experience.

## Conclusion

Detection of specific needs or the most appropriate approach for patient-specific support is often dependent on the personal sensitivity of the healthcare professional. This study applied the SHE model to recognise and understand the individual supportive care needs of people with IMIDs. Strikingly, the model proved applicable across all six diseases studied.

The findings enabled the differentiation of multiple forms of supportive care across the SHE Segments, providing healthcare professionals with evidence-based guidance to tailor individualised treatment approaches. Despite differences in diagnosis, patients displayed notable similarities in characteristics, behaviours, and needs. Consequently, the *what* (types of supportive care offered) and the *how* (methods of delivery) were also largely consistent across conditions. This supports the development of a supportive care design that transcends disciplines and care levels.

By segmenting patients according to health perception, the SHE model offers a practical framework for integrated care delivery that clarifies provider roles, enhances continuity, and fosters interdisciplinary alignment. The results also suggest considerable added value for improving care coordination, thereby increasing patients’ perceived continuity of care.

The core elements of effective supportive care identified in this study—attention, acknowledgement, and active listening—are critical for delivering patient-centred, appropriate care. Embedding these principles within multidisciplinary teams, supported by the structured use of the SHE model, has the potential to improve both the quality and efficiency of care. Future implementation and evaluation in diverse clinical contexts will be essential to fully realise its benefits.

## Data Accessibility Statement

The raw data used during this study is not publicly available, but is available from the corresponding author upon reasonable request.

## Figures and Tables

**Table:** 

HS	Hidradenitis Supperativa
IBD	Inflammatory Bowel Disease
IMIDs	Immune-mediated inflammatory disorders
Non-WMO	Not subject to the Medical Research Involving Human Subjects Act
PROMs	Patient Reported Outcome Measures
PsO	Psoriasis
QoL	Quality of Life
RA	Rheumatoid Arthritis
SHE	Subjective Health Experience
SpA	Spondylarthritis
